# Modeling the Impact of Interventions on an Epidemic of Ebola in Sierra Leone and Liberia

**DOI:** 10.1371/currents.outbreaks.4d41fe5d6c05e9df30ddce33c66d084c

**Published:** 2014-11-06

**Authors:** Caitlin M. Rivers, Eric T. Lofgren, Madhav Marathe, Stephen Eubank, Bryan L. Lewis

**Affiliations:** Network Dynamics and Simulation Science Laboratory, Virginia Bioinformatics Institute, Virginia Tech, Blacksburg, Virginia, USA; Network Dynamics and Simulation Science Laboratory, Virginia Bioinformatics Institute, Virginia Tech, Blacksburg, Virginia, USA; Network Dynamics and Simulation Science Laboratory, Virginia Bioinformatics Institute, Virginia Tech, Blacksburg, Virginia, USA; Network Dynamics and Simulation Science Laboratory, Virginia Bioinformatics Institute, Virginia Tech, Blacksburg, Virginia, USA; Network Dynamics and Simulation Science Laboratory, Virginia Bioinformatics Institute, Virginia Tech, Blacksburg, Virginia, USA

**Keywords:** disease model, ebola, ebolavirus

## Abstract

Background: An Ebola outbreak of unparalleled size is currently affecting several countries in West Africa, and international efforts to control the outbreak are underway. However, the efficacy of these interventions, and their likely impact on an Ebola epidemic of this size, is unknown. Forecasting and simulation of these interventions may inform public health efforts. Methods: We use existing data from Liberia and Sierra Leone to parameterize a mathematical model of Ebola and use this model to forecast the progression of the epidemic, as well as the efficacy of several interventions, including increased contact tracing, improved infection control practices, the use of a hypothetical pharmaceutical intervention to improve survival in hospitalized patients. Findings: Model forecasts until Dec. 31, 2014 show an increasingly severe epidemic with no sign of having reached a peak. Modeling results suggest that increased contact tracing, improved infection control, or a combination of the two can have a substantial impact on the number of Ebola cases, but these interventions are not sufficient to halt the progress of the epidemic. The hypothetical pharmaceutical intervention, while impacting mortality, had a smaller effect on the forecasted trajectory of the epidemic. Interpretation: Near-term, practical interventions to address the ongoing Ebola epidemic may have a beneficial impact on public health, but they will not result in the immediate halting, or even obvious slowing of the epidemic. A long-term commitment of resources and support will be necessary to address the outbreak.

## Introduction

West Africa is currently experiencing an unprecedented outbreak of Ebola, a viral hemorrhagic fever. On March 23, 2014 the World Health Organization announced through the Global Alert and Response Network that an outbreak of Ebola virus disease in Guinea was unfolding^1^^,^ 2 ^,^ 3. Ebola is generally characterized by sporadic, primarily rural outbreaks, and has not been seen before in West Africa, or in an outbreak of this size.

As of October 5, 2014, the World Health Organization has reported 8,033 cases of Ebola virus disease in Sierra Leone, Liberia, Guinea, Nigeria and Senegal, with sporadic cases occurring outside West Africa^4^. Considerable attention has been focused on preventing the outbreak from spreading further, either within Africa or intercontinentally. In principle many of the measures to contain the spread of Ebola, such as intensive tracing of anyone in contact with an infected individual and the use of personal protective equipment (PPE) for healthcare personnel treating infected cases, are straightforward^5^. However, implementing those interventions in a resource poor setting in the midst of an ongoing epidemic is far from simple, and subject to a great deal of uncertainty.

Mathematical models of disease outbreaks can be helpful under these conditions by providing forecasts for the development of the epidemic that account for the complex and non-linear dynamics of infectious diseases and by projecting the likely impact of proposed interventions before they are implemented. This in turn provides policy makers, the media, healthcare personnel and the public health community with timely, quantifiable guidance and support^6^^,^ 7 ^,^ 8 ^,^ 9.

We use a mathematical model to describe the development of the Ebola outbreak to date, provide short term projections for its future development, and examine the potential impact of several interventions, namely increased contact tracing, improved access to PPE for healthcare personnel, and the use of a pharmaceutical intervention to improve survival in hospitalized patients.

## Methods

Outbreak Data

A time series of reported Ebola cases was collected from public data released by the World Health Organization, as well as the Ministries of Health of the afflicted countries. These data sets do not include patient-level information, but rather laboratory confirmed, suspected or probable cases of the disease, which is thought to represent the best available estimate of the current state of the epidemic. A curated version of this data is available at https://github.com/cmrivers/ebola.

A compartmental model was used to describe the natural history and epidemiology of Ebola, adapted from Legrand *et al.* 10 which was previously used to describe the 1995 Democratic Republic of Congo and 2000 Uganda Ebola outbreaks. Briefly, the population is divided into six compartments, as shown in Figure 1. Susceptible individuals (S) may become Exposed (E) after contact with an infectious individual and transition in turn to the Infectious (I) class after the disease’s incubation period, thereafter capable of infecting others. A proportion of these individuals may be Hospitalized (H). Both untreated patients in I and hospitalized patients in H may experience one of two outcomes: patients may die, with a chance of infecting others during the resulting funeral (F) before being removed from the model (R), or they may recover, at which point they are similarly removed. The system of ordinary differential equations describing this model is below, and a technical note on the system is available in Appendix 1.

**Compartmental flow of a mathematical model of the Ebola Epidemic in Liberia and Sierra Leone, 2014.**
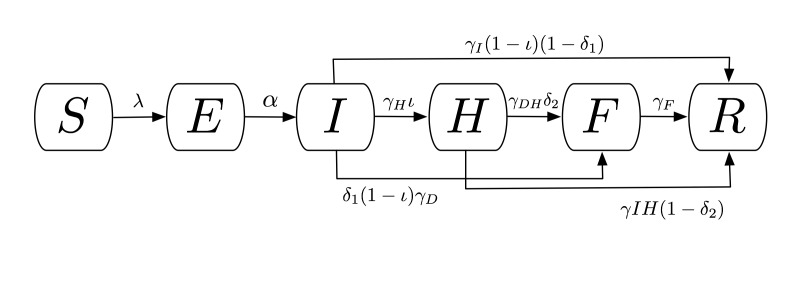
The population is divided into six compartments: Susceptible (S), Exposed (E), Infectious (I), Hospitalized (H), Funeral (F) – indicating transmission from handling a diseased patient’s body, and Recovered/Removed (R). Arrows indicate the possible transitions, and the parameters that govern them. Note that λ is a composite of all β transmission terms described in Table 1.


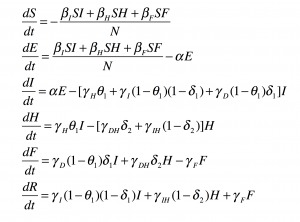


Model Fitting and Validation

A deterministic version of the model was fit and validated to the current outbreak data using least-squares optimization, with seed values from the Uganda outbreak described in Legrand *et al* 10 . The last 15 days of reported cases were given one-quarter of the weight in the model to preferentially fit the most recent data. Based on anecdotal reports from the field (unpublished), candidate optimized fits were accepted such that roughly one-quarter of infections each came from contacts with hospitalized patients or funereal transmission, with the balance being from person-to-person spread within the community. The optimizer was further constrained to plausible parameter values, such as an upper bound of 20 days for infection duration, and 0 to 1 for probabilities or proportions. This model was fit only for Sierra Leone and Liberia, as the outbreak in Guinea has a unique epidemic curve which necessitates an alternative model design beyond the scope of this paper. The fitted parameters for this model, as well as their descriptions, may be found in Table 1.

This validated model provides a mathematical description of the epidemic up to the present. In order to forecast into the future, a stochastic version of the model was implemented using Gillespie’s algorithm with a tau-leaping approximation^11^^,^ 12, which treats individuals as discrete units and converts the deterministic rates in the calibration model into probabilities, allowing random chance to come into play. Using the parameters from the calibration model, as well as the number of individuals in each compartment at the present date, 250 simulations of this model were run until December 31, 2014, giving a fan of potential epidemic trajectories that accounts for uncertainty in the forecast due to chance^13^. All models were implemented in Python 2.7, and the stochastic simulations used the StochPy library^14^ .

Modeled Interventions

Based on interventions that are technically, but not necessarily socially, feasible in the foreseeable future, we model five scenarios to examine their likely impact on the development of the epidemic. First, we model improved contact tracing by increasing the fraction of infected cases that are diagnosed and hospitalized from the baseline scenario of 51% in Liberia and 58% in Sierra Leone to 80%, 90% and 100%, and a concordant decrease in the time it takes for an infected individual to be hospitalized by 25%. This scenario could also be considered to represent improved access to healthcare, or improved public support for the hospitalization of sick individuals. Second, we explore the impact of simultaneously (1) decreasing the contact rate for hospitalized cases (β_H_) to represent the increased use of PPE as supplies and awareness of the outbreak increase as well as (2) eliminating the possibility of post-mortem infection from hospitalized patients due to inappropriate funereal practices. Third, we model both a simultaneous (1) decrease in β_H_ (lack of post-mortem infection from hospitalized cases) and (2) increase in the proportion of hospitalized cases. This models the effect of a joint, intensified campaign to identify and isolate patients – the conventional means of containing an Ebola outbreak – with the necessary supplies and infrastructure to treat these patients using appropriate infection control practices. Finally, we model a pharmaceutical intervention that increases the survival rate of hospitalized patients by 25%, 50% and 75%, with a moderately high level of contact tracing (80%).

**Model Parameters and Fitted Values for a Model of an Ebola Epidemic in Liberia and Sierra Leone, 2014.** 

Parameter	Liberia Fitted Values	Sierra Leone Fitted Values
Contact Rate, Community (β_I_)	0.160	0.128
Contact Rate, Hospital (β_H_)	0.062	0.080
Contact Rate, Funeral (β_F_)	0.489	0.111
Incubation Period (1/α)	12 days	10 days
Time until Hospitalization (1/γ_H_)	3.24 days	4.12 days
Time from Hospitalization to Death (1/γ_DH_)	10.07 days	6.26 days
Duration of Traditional Funeral (1/γ_F_)	2.01 days	4.50 days
Duration of Infection (1/γ_I_)	15.00 days	20.00 days
Time from Infection to Death (1/γ_D_)	13.31 days	10.38 days
Time from Hospitalization to Recovery (1/γ_IH_)	15.88 days	15.88 days
Fraction of Infected Hospitalized (θ_1_)	0.197	0.197
Case Fatality Rate, Unhospitalized (δ_1_)	0.500	0.750
Case Fatality Rate, Hospitalized (δ_2_)	0.500	0.750

Human Subjects

As this study uses publicly available data without personal identifiers, it was determined not to require IRB approval.

## Results

Model Fit and Prediction

The deterministic model fit well for both Liberia and Sierra Leone, with the predicted curve of cumulative cases following the reported number of cases in both countries. The end-of-year forecast shows a range of uncertainty for each country of several thousand cases between the most optimistic and pessimistic scenarios. However, the number of cumulative cases is forecast to continue rising extremely rapidly, with the bulk of the epidemic yet to come (Figure 2). This suggests an extremely poor outlook for the course of the epidemic without intensive interventions.

In the baseline end-of-year forecasts for both Sierra Leone and Liberia, person-to-person transmission within the community made up the bulk of transmission events, with a median (IQR: Interquartile Range) of 117,877 (115,100– 120,585) cases arising from the community in the Liberia forecast and 30,611 (29,667 – 31,857) in the forecast for Sierra Leone. Both had fewer hospital transmissions – 21,533 (21,025 – 21,534) in Liberia and 5,474 (5,306 – 5,710) in Sierra Leone, than transmissions arising from funerals – 35,993 (35,163 – 36,789) in Liberia and 9,768 (9,470 – 10,137) in Sierra Leone. For brevity, only the results of the Liberia model are reported below, with the results from Sierra Leone in the electronic supplement. The epidemic trajectories for all modeled interventions may also be found in the Web Material.

**Fitted Compartmental Model for Ebola Epidemic in Liberia and Sierra Leone, 2014, with 250 Iterations of a Stochastic Forecast to December 31, 2014.**
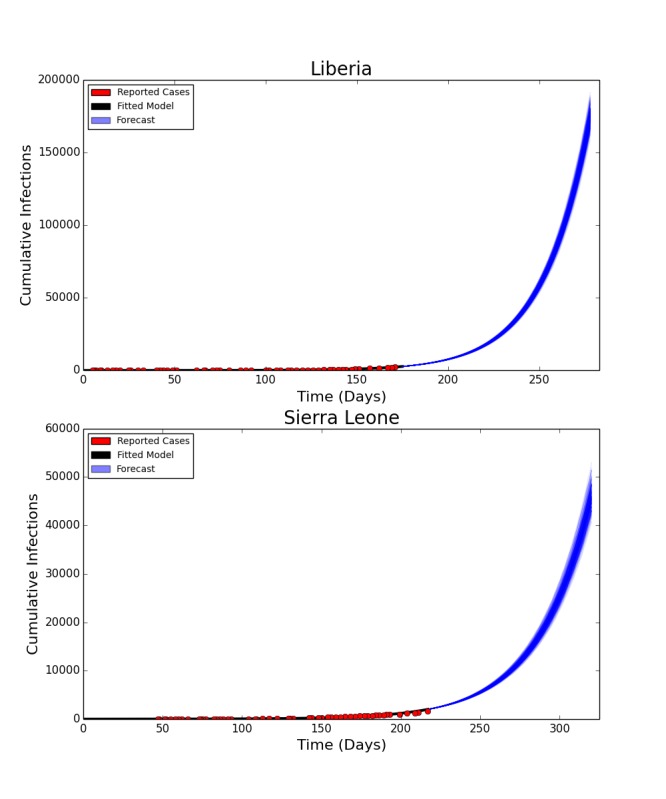
Red dots depict the reported number of cumulative cases of Ebola in each country, with the black line indicating the deterministic model fit. Each blue line indicates one of two hundred and fifty stochastic simulated forecasts of the epidemic, with areas of denser color indicating larger numbers of forecasts.

Basic Reproduction Number

The basic reproduction number (R_0_) for the baseline scenario was calculated in the same manner as in Legrand *et al.* 10 Briefly, R_0_ is broken into three components, representing the respective contributions of community, hospital and funereal transmissions, as well as an overall R_0_ reflecting the epidemic potential for the disease. In the baseline scenario, we estimate an overall R_0_ of 2.22, made up of an R_0 _of 1.35 from the community, 0.35 from hospitals and 0.53 from funerals for Liberia. Sierra Leone's R_0_ was estimated to be 1.78, made up of an R_0_ of 1.11 from the community, 0.24 from hospitals and 0.43 from funerals. These estimates are similar to estimates for the current outbreak reported elsewhere. For brevity, only the overall R_0_ estimates will be reported here. The breakdown of R_0 _by source can be found in the Figshare Web Material.

Intensified Contact Tracing and Infection Control

The forecasted distribution of cases under intensified contact tracing is shown in Figure 3. There is a shift from community transmission toward hospital transmission, though at extremely high levels of contact tracing and hospitalization, the impact of the intervention on the course of the outbreak also results in fewer hospitalized cases. There is a less pronounced but still substantial downward shift in funeral cases, and a decrease in total cases in Liberia. This decrease is not sufficient to shift the cumulative case curve off its steep upward trajectory, but only lessens its magnitude. The 80%, 90% and 100% of patients traced and hospitalized scenarios resulted in an overall reduction of R_0_ to 2.11, 2.01 and 1.89 respectively.

**Distribution of Forecast Cases of Community, Hospital, Funeral and Total Cases for Ebola Epidemic, Liberia, 2014, at Baseline, 80%, 90% and 100% of Patients Traced and Hospitalized.**
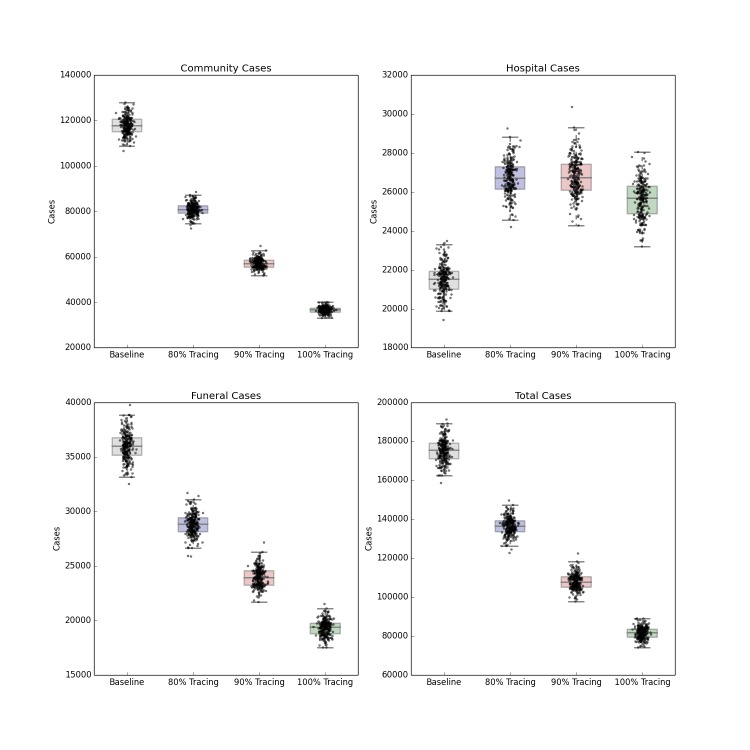
Box plots depict the median, interquartile range and 1.5 times the interquartile range for each scenario. Each individual simulated forecast is shown as a single dot, jittered so as to depict the complete distribution of the data.

The improved infection control scenario decreased β_H _to represent decreased risk of hospital transmission due to increased PPE, increased number of healthcare workers or greater awareness of the epidemic resulting in greater care while treating patients with undiagnosed febrile illness. Additionally, it eliminated the potential for post-mortem transmission during the funereal process. This combination of interventions resulted in a marked decrease in the overall number of cases (Figure 4), and a reduction of R_0_ to 2.13, 2.05 and 1.96 for 25%, 50% and 75% reductions in the hospital transmission contact rates and improvements in the disposal of the remains of Ebola victims.

**Distribution of Forecast Cases of Community, Hospital, Funeral and Total Cases for Ebola Epidemic, Liberia, 2014, at Baseline, 25%, 50% and 75% Reductions in Hospital Transmission Contact Rates (βH).**
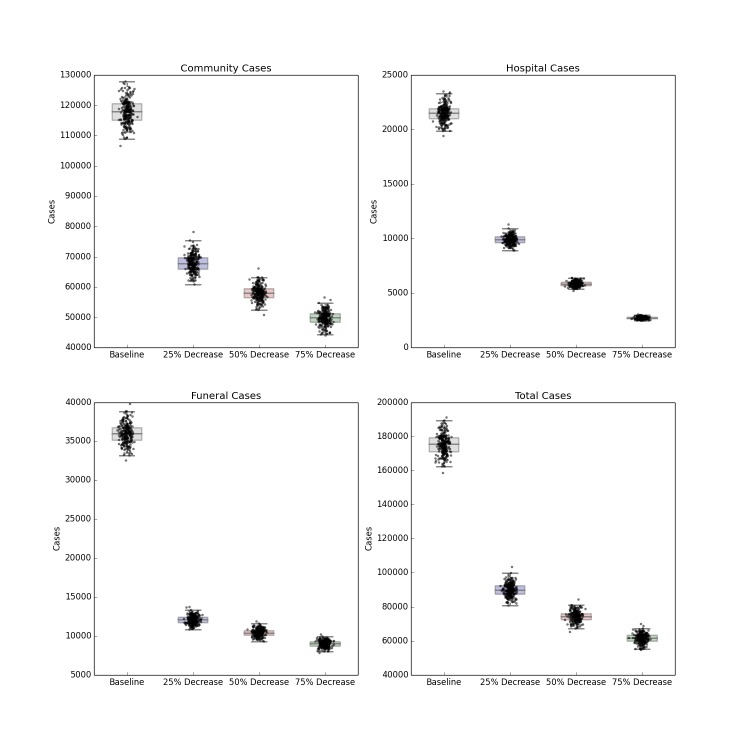
Box plots depict the median, interquartile range and 1.5 times the interquartile range for each scenario. Each individual simulated forecast is shown as a single dot, jittered so as to depict the complete distribution of the data.

Major reductions in all sources of cases were seen, with the most dramatic drop in the relative number of cases arising from the reduction of within-hospital transmissions. However, even with substantially reduced transmission and a decrease in the burden of mortality from the outbreak, improved infection control was also insufficient to push the epidemic off its steep upward trajectory.

Figure 5 shows the median decrease in cases as compared to baseline for simulations combining increased contact tracing and a reduction in the risk of hospital transmission from those who are isolated and treated. The most optimistic of these scenarios, with complete contact tracing and a 75% reduction in hospital transmission results in more than 165,000 fewer total cases over the course of the forecasted period, as compared to the baseline scenario (Figure 6). The overall R_0_ in this scenario is reduced to 1.72. This represents a ten-fold reduction in the number of cases, and is a major improvement epidemic trajectory. However even under this scenario, the epidemic is slowed and mitigated, rather than fully stopped, with transmission still ocurring after the end of the year.

**Distribution of Forecasted Cases of Community, Hospital, Funeral and Total Cases for Ebola Epidemic, Liberia, 2014, at Baseline, 25%, 50% and 75% Reductions in Hospital Transmission Contact Rates (βH) with 80%, 90% and 100% of Patients Traced and Hospitalized.**
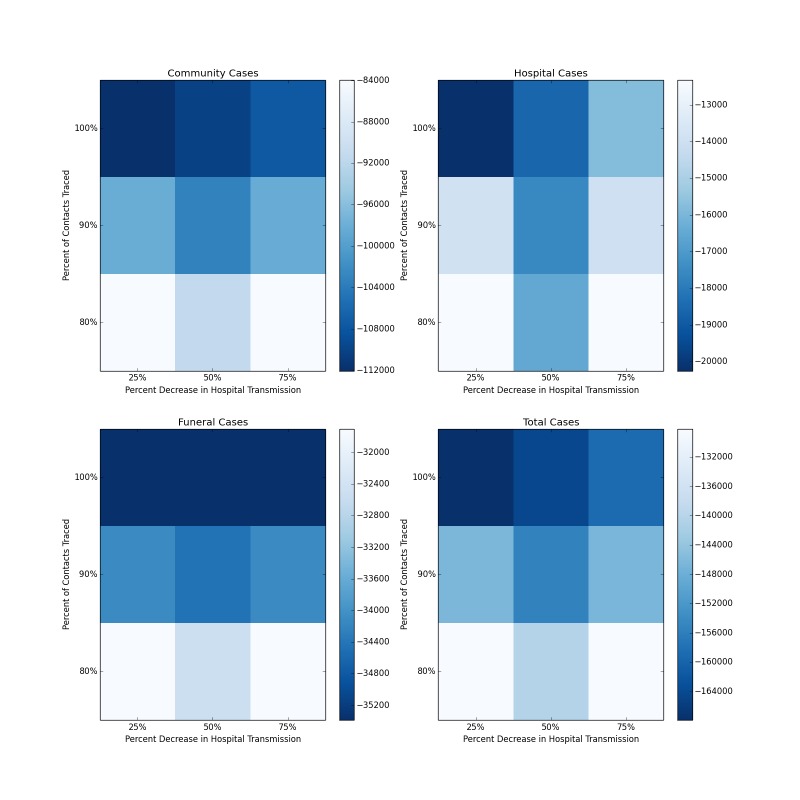
Each box represents the median result of 250 forecasted epidemics, each with a % of contacts traced and a % decrease in hospital transmission. Areas of deeper blue indicate progressively greater reductions of the median number of cases.

**Forecasted Cumulative Cases for Ebola Epidemic, Liberia, 2014 with 75% Reduction in Hospital Transmission Contact Rates (βH) with 100% of Patients Traced and Hospitalized.**
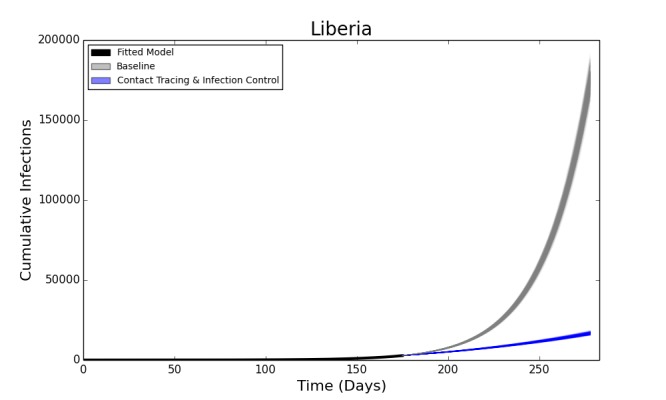
The solid black line represents the deterministic model fit of the epidemic to present, with each grey line representing a single simulated forecast with no interventions in place, and each blue line representing a single simulated forecast of the epidemic with 100% of contacts traced, a 75% reduction in hospital transmission (βH) and no post-mortem infections from hospitalized patients. Areas of darker color indicate more forecasts with that result.

Increased Availability of Pharmaceutical Interventions

The introduction of a pharmaceutical intervention that dramatically improves the survival rate of hospitalized patients also leads to a less severe outbreak, shown in Figure 7. Compared to contact tracing alone, there is a small reduction in the number of hospitalized cases (as the scenario implies no change in infection control practices), but a stronger decrease in the number of community, funeral and overall cases depending on the efficacy of the hypothetical pharmaceutical. An efficacy that reduces the case fatality rate of hospitalized patients by 25%, 50% or 75% results in a corresponding reduction of R_0_ to 2.03, 1.94 and 1.85 respectively. As with the other forecasts above, this intervention also fails to halt the progress of the epidemic, though it does considerably reduce the burden of disease.

**Distribution of Forecast Cases of Community, Hospital, Funeral and Total Cases for Ebola Epidemic, Liberia, 2014, at Baseline, 25%, 50% and 75% Reductions in Case Fatality Rate Due to a Hypothetical Pharmaceutical Intervention.**
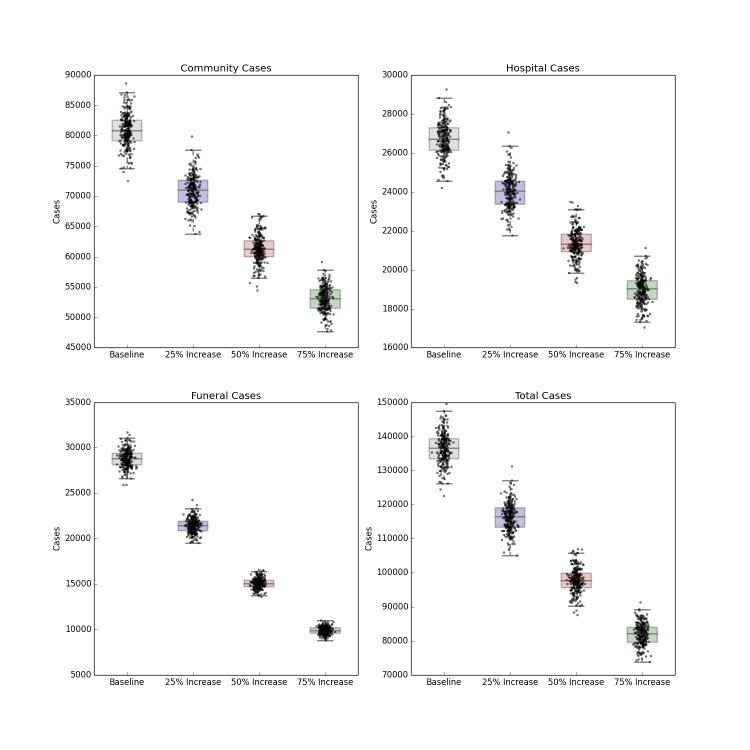
Box plots depict the median, interquartile range and 1.5 times the interquartile range for each scenario. Each individual simulated forecast is shown as a single dot, jittered so as to depict the complete distribution of the data.

## Discussion

The control of Ebola outbreaks in the past has been a straightforward, albeit difficult application of infection control and quarantine policies. In principle, these types of interventions should be applicable to this outbreak as well. However, it remains unclear whether they can be implemented at the unprecedented scale of the current outbreak. This study attempts to address whether or not aggressive interventions could arrest, or at least mitigate, the epidemic.

Our findings suggest that, for at least in the near term, some form of coordinated intervention is imperative. The forecasts for both Liberia and Sierra Leone in the absence of any major effort to contain the epidemic paint a bleak picture of its future progress, which suggests that we are in the opening phase of the epidemic, rather than near its peak. These findings are in line with predictions from other models which, despite using different methods and different data sources, have all estimated similar basic reproductive numbers, and forecast that the epidemic is currently beyond the point where it can be easily controlled^9^^,^ 15 ^,^ 16.

Of the modeled interventions applied to the epidemic, the most effective by far is a combined strategy of intensifying contact tracing to remove infected individuals from the general population and placing them in a setting that can provide both isolation and dedicated care. This intervention requires that clinics have the necessary supplies, training and personnel to follow infection control practices. Although both of these interventions in isolation also have an impact on the epidemic, they are much more effective in parallel. In particular, the slight increase in cases in Sierra Leone at the lower end of the modeled contact tracing range, when unaccompanied by a concordant increase in infection control, highlights the necessity of these two interventions being implemented side-by-side.

The hypothetical mass application of a novel pharmaceutical like the one administered to two American aid workers^17^^,^ 18 had a much smaller impact on the course of the epidemic itself. While certainly lessening the burden of mortality of those infected (in the most optimistic scenario modeled, reducing the case-fatality rate from 50% to 12·5%), the downstream effects of such an intervention are relatively minor, as there is no suggestion that any candidate treatments have a substantial impact on transmission. This will impact not only the evaluation of those treatments, which should thus focus primarily on their patient-level efficacy, but also in communicating to the media and the public that, despite the use of these drugs, the benefits that will be seen from them are in decreases to mortality due to infection, not in a halt to the epidemic itself.

Despite the considerable impact the proposed interventions have on the burden of disease, none of them are forecast, at least in the short term, to halt the epidemic entirely. It is possible these interventions will have a longer-term impact on the epidemic, however we have avoided projecting out further than the end of the year due to the inherent uncertainty in an emerging epidemic. This in turn suggests another communication challenge for public health planners. While all of the proposed interventions are worth pursuing, and will have an impact on the epidemic and public health, the attention of the international community must be sustained in the long term in order to ensure the necessary supplies and expertise remain present in the affected areas. Additionally, in light of public resistance to the limited types of these interventions already in place, public awareness and acceptance of intensified interventions must be built, as there will not be an immediate cessation to the epidemic, or even necessarily a clear sign that the situation is improving.

This study is not without limitations. As with all mathematical models, the results of the study depend on the assumptions about the natural history of Ebola, its epidemiology, and the values of the parameters used – as well as the quality of the data used to fit the model. Particularly, this model is validated against data that records cases on their time of reporting, rather than their time of onset, so the model time series may be shifted by several days. Because of the relatively small number of historical Ebola outbreaks, the unusual size of this outbreak, and the difficulty collecting data during an emerging epidemic, the accuracy of this model and its parameters is difficult to ascertain. It does however represent our best understanding of the epidemic using available data, and the model has proven capable of predicting the ongoing development of the epidemic, as well as having been used to model previous Ebola outbreak. The uncertainty inherent in model prediction has been addressed with the short forecasting window, and with the use of stochastic simulation to aid in quantifying uncertainty inherent within the model system.

The ongoing Ebola epidemic in West Africa demands international action, and the results of this study support that many of the interventions currently being implemented or considered will have a positive impact on reducing the burden of the epidemic. However, these results also suggest that the epidemic has progressed beyond the point wherein it will be readily and swiftly addressed by conventional public health strategies. The halting of this outbreak will require patient, ongoing efforts in the affected areas and the swift control of any further outbreaks in neighboring countries.

## Figures and Tables

**Figure:**
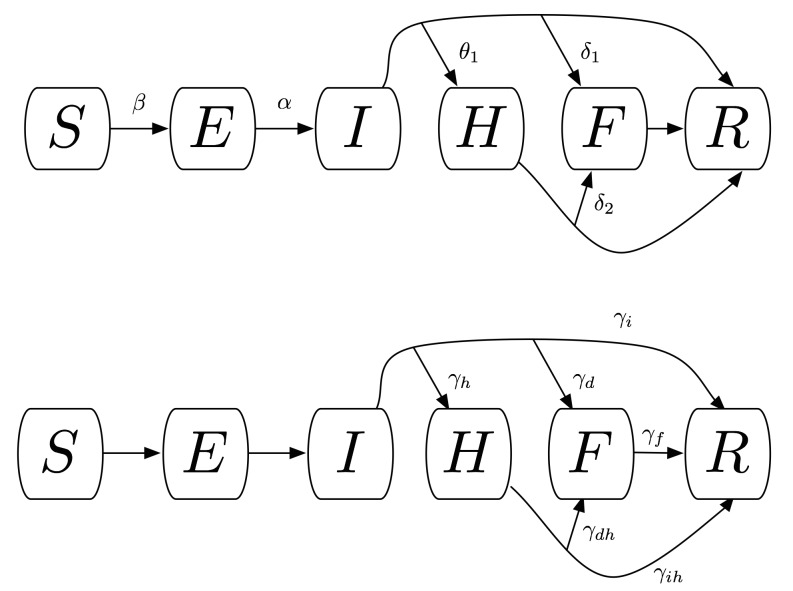
**Appendix Figure 1.** The fractions of people distributed across different paths through the compartments (top) and their respective transition rates (bottom).

**Figure:**
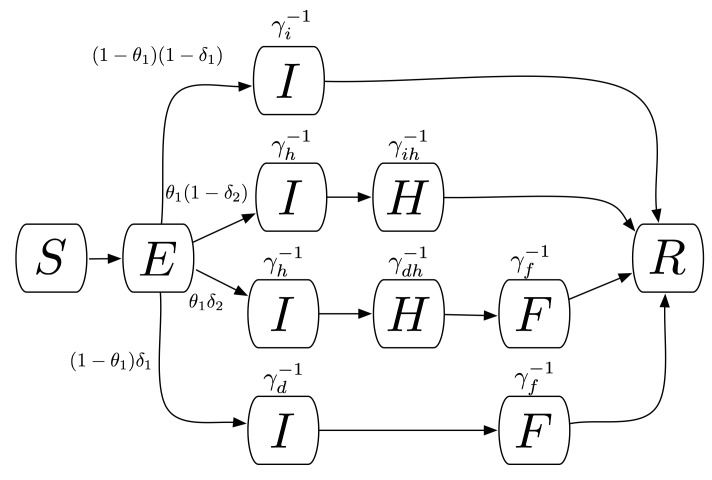
**Appendix Figure 2.** Expanded view of the model used in this manuscript, with each path labeled by its overall probability and the transition rates represented by the mean residence times associated with each compartment in each path.

## Appendix 1

**Technical Note on ‘Modeling the Impact of Interventions on an Epidemic of Ebola in Sierra Leone and Liberia’ **

Based on comments from readers of the manuscript, we believe it is valuable to explore the way multiple competing pathways for an individual within Legrand et al’s model, and by extension the model used in this paper, are handled. In a generic SEIR model, the system of differential equations is given by: 

\begin{equation*}\[\begin{array}{l}\%0A\frac{{dS}}{{dt}} =  - \beta SI\\\%0A\frac{{dE}}{{dt}} = \beta SI - \gamma E\\\%0A\frac{{dI}}{{dt}} = \gamma E - \delta I\\\%0A\frac{{dR}}{{dt}} = \delta I\%0A\end{array}\]\end{equation*}

This can be thought of as a deterministic (or stochastic, when simulated using a stochastic simulation algorithm) version of a Markov process, moving people from E to I and I to R with the corresponding probabilities on each step. In this interpretation, the distribution for residence time in each state is exponential, with means of Υ^-1^ and δ^-1^ respectively.

Because the model used in this manuscript has multiple potential transitions for two of the compartments (I and H), it uses a somewhat more complex transition scheme that is not as intuitive. Rather than the transition between two states being a single process governed by a single residence time parameter, the people transitioning out of a compartment are split into the fractions following each potential “path” out of that compartment, each with its own corresponding residence time. This is shown below in Appendix Figure 1 with both processes broken out, and Appendix Figure 2 with the processes shown together.

In effect, while technically within the same compartment, patients who will experience different outcomes are treated as being on distinct, independent pathways. This is a conceptually different process than individuals all having the possibility of each outcome, and transitioning between them based entirely on waiting times. As such, the mean residence times estimated by the model are not weighted means, but the means for each particular path, where the “outcome” of that path is already known. The composition of a system of equations that does provide weighted means is relatively straightforward. However, the authors have elected to use the same system as Legrand et al. to maintain consistency with much of the existing Ebola literature. 
